# Dietary transformations and health implications in migrant populations: a global perspective

**DOI:** 10.3389/fnut.2025.1623556

**Published:** 2025-08-18

**Authors:** Joseph Vinod Varre, Mia Dustin, Stephan Van Vliet

**Affiliations:** Department of Nutrition, Dietetics, and Food Sciences, Utah State University, Logan, UT, United States

**Keywords:** dietary acculturation, migration, nutrition transition, migrant health, chronic disease risk, food environment, socioeconomic determinants, gut microbiota

## Abstract

**Background:**

Dietary acculturation—the process by which migrants adopt the dietary patterns of their host country—has become increasingly relevant given the unprecedented scale of international migration. This phenomenon is often associated with a shift from traditional diets toward host-country patterns that are higher in ultra processed foods, added sugars, and fats, with potential implications for chronic disease risk.

**Objective:**

This mini-review aims to synthesize global evidence on the dietary transformations experienced by migrant populations and to assess the health implications and modulating factors influencing these changes.

**Methods:**

A targeted literature search was conducted in PubMed, Scopus, and Web of Science for articles published between January 2000 and April 2024, using keywords related to dietary acculturation, migration, and health outcomes. After applying inclusion and exclusion criteria, 30 studies directly addressing dietary change and health outcomes post-migration were included. Key themes were identified through iterative synthesis.

**Results:**

Evidence indicates a consistent trend of dietary acculturation across diverse migrant groups, typically involving increased consumption of energy-dense, processed foods (a 15–20% increase) and decreased intake of traditional staples such as whole grains (down by 10–15%), pulses, and fresh vegetables. These dietary shifts are associated with a heightened risk of obesity (increasing by 5–10%), type 2 diabetes (7–12% rise), and cardiovascular diseases. Factors such as length of residence (1–5 years), age at migration (20–30 years), socioeconomic status (bottom 20%), food environment (availability dropping by 30%), and health literacy significantly modulate these changes. Emerging evidence also points to changes in gut microbiota as a consequence of dietary transformation.

**Conclusion:**

Dietary acculturation among migrant populations is a multifaceted process that increases the risk of nutrition-related chronic diseases. There is a critical need for culturally sensitive public health interventions and policies that support the preservation of healthy traditional diets while facilitating healthy adaptation to new food environments. Addressing research gaps—such as longitudinal data and the experiences of underrepresented migrant groups—will strengthen strategies to mitigate adverse health outcomes.

## Introduction

**Figure 1:**
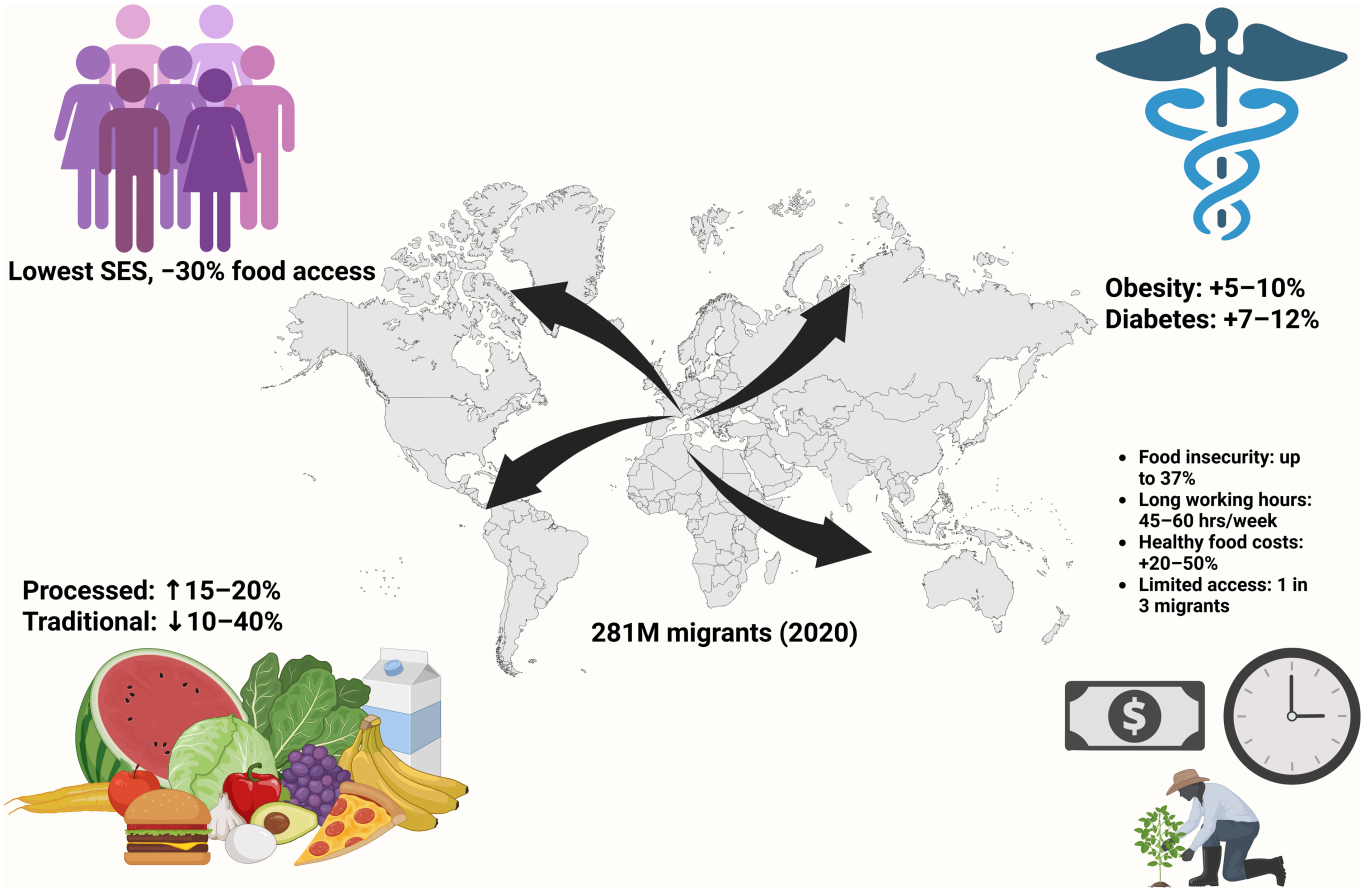
Conceptual framework illustrating the drivers and consequences of dietary acculturation in migrant populations. SES, socioeconomic status. Created in BioRender. Van Vliet, S. (2025) https://BioRender.com/ghtni6q.

International migration is a complex process affecting individuals’ daily lives, social structures, and health behaviors, including dietary patterns. As of 2020, over 281 million people lived outside their country of birth, underscoring the global relevance of understanding how migration influences health and nutrition (1, 2). A consistent finding across epidemiological and public health research is that dietary habits often undergo substantial transformation following migration. Migrants frequently shift from traditional diets—rich in whole grains, legumes, fruits, and vegetables—to eating patterns characterized by higher intakes of processed foods, added sugars, and saturated fats (2, 3, 4). For example, some migrant groups experience a 20–40% reduction in the consumption of traditional staples and a 30% increase in processed food intake within a decade of resettlement (2, 4, 5). These changes are frequently linked to increased rates of obesity, type 2 diabetes, and cardiovascular disease; prevalence rates for these conditions are 1.5 to 2 times higher among migrants after acculturation compared to their pre-migration status or host-country natives (6, 7). The drivers of these dietary shifts are multifactorial and include socioeconomic constraints, the food environment, acculturation pressures, and changes in time allocation and occupational demands (2). Figure 1 presents a conceptual framework illustrating how demographic factors, health outcomes, the food environment, and socioeconomic factors interact to shape dietary transitions and health risks among migrant populations.

Despite the growing body of literature, significant research gaps remain, including a lack of longitudinal data, limited representation of refugees and undocumented migrants, and insufficient focus on structural determinants such as food security and health literacy (8–11). Most existing reviews focus on high-income host countries, leaving a knowledge gap regarding dietary acculturation in low- and middle-income settings and among diverse migration contexts (6, 9, 12, 13). To address these limitations, future research should adopt interdisciplinary approaches, integrate qualitative and quantitative data, and employ community-based participatory research methods to develop culturally tailored interventions that promote healthy eating behaviors among migrant populations. Dietary acculturation has significant impacts on migrant health, with ultra processed food adoption driving increased chronic disease risk (14). The objective of this mini-review is to synthesize current global evidence on the patterns and determinants of dietary acculturation among migrant populations. By integrating data across continents, migrant groups, and study designs, this review highlights the health consequences of dietary transitions, identifies key modulating factors, and discusses emerging areas such as gut microbiota and mental health. The review also provides a conceptual framework to guide future research and inform culturally sensitive public health interventions aimed at preserving healthy dietary practices in migrant communities.

## Methods

A focused review of existing literature was undertaken to consolidate evidence pertaining to dietary acculturation and its effects on the health of migrant populations. Searches were conducted in PubMed, Scopus, and Web of Science for relevant articles published between January 2000 and April 2024. The search strategy incorporated combinations of terms such as “dietary acculturation,” “migration,” “nutrition transition,” “migrant diet,” “traditional foods,” “health outcomes,” “chronic disease,” “refugees,” and “immigrants.” Approximately 200 article titles and abstracts were initially assessed for relevance. Studies were included if they investigated dietary changes occurring among migrants or refugees post-migration and reported associations with health outcomes, including obesity, diabetes, cardiovascular disease, or food insecurity. Studies were excluded if they exclusively examined pre-migration dietary habits, involved non-human subjects, or lacked original data or review content pertinent to acculturation and health. Following a thorough full-text review and the application of these criteria, 30 articles were selected for in-depth synthesis. To establish a conceptual framework, additional resources were consulted, including models describing dietary acculturation processes and dietary patterns prevalent in host countries. The reference lists of selected articles were also reviewed to identify additional relevant studies. Key themes—such as dietary transformations, modulating factors, health implications, and structural determinants—were identified through an iterative process based on recurring concepts within the selected literature. The synthesis emphasized quantitative data and comparative results across different migrant groups whenever feasible.

### Study selection flow

**Figure 2:**
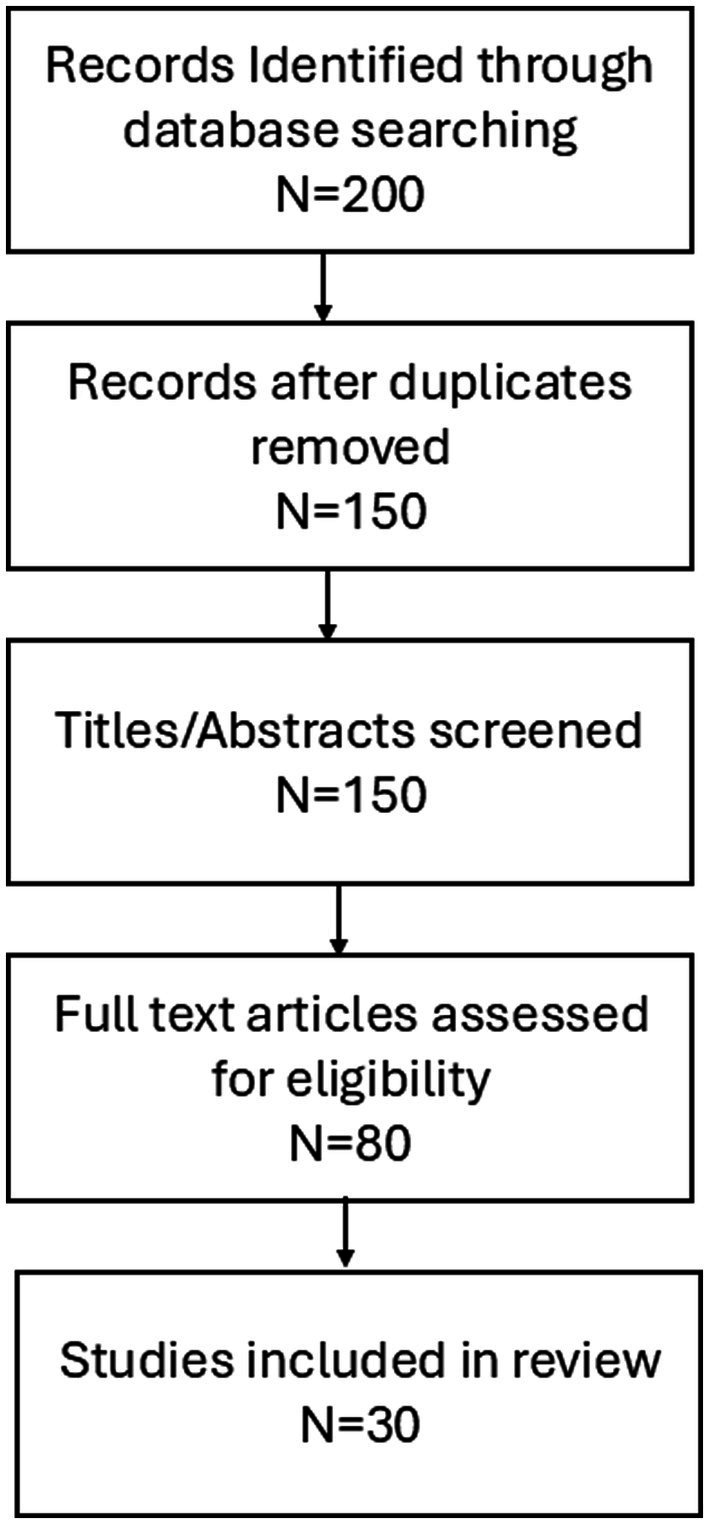
Research process overview.

A total of 200 records were identified through database searching. After removal of duplicates, 150 unique records remained for screening. Titles and abstracts were screened, resulting in 80 full-text articles assessed for eligibility. After applying inclusion and exclusion criteria, 30 studies were included in the final synthesis (Figure 2).

### Dietary transformations and modulating factors

#### Global patterns of dietary acculturation

Dietary acculturation among migrant populations typically involves a shift from traditional diets—rich in whole grains, legumes, fruits, and vegetables—to host-country patterns characterized by higher intakes of processed foods, refined sugars, and saturated fats (15). Across diverse cultural and geographic contexts, consistent patterns of dietary transformation have been documented among migrant groups; these shifts commonly involve the displacement of traditional dietary staples with energy-dense, micronutrient-poor alternatives readily available in the host country (2, 4). For instance, South Asian migrants in Europe tend to increase their consumption of Ultraprocessed meat and dairy products while decreasing their carbohydrate intake and switching from whole grains to refined sources, leading to lower fiber intake (4, 16). Similar trends have been observed among Latin American migrants in the United States, where traditional diets high in fruits, vegetables, and legumes are replaced by diets high in processed foods, sugary beverages, and ultra processed animal products (17, 18). Furthermore, the transition often includes increased consumption of fast foods and sweetened beverages, coupled with reduced intake of traditional dishes prepared at home. The impact of dietary acculturation extends beyond individual food choices, influencing overall meal patterns and eating behaviors (19).

Dietary acculturation encompasses various dimensions, including changes in food choices, preparation methods, meal timing, and eating behaviors, as well as a decline in the consumption of traditional foods (20). This multifaceted process is shaped by factors such as the availability and accessibility of traditional foods in the host country, socioeconomic status, cultural identity, and social networks. Despite the widespread acknowledgement of dietary acculturation, growing evidence suggests that the relationship between dietary habits, immigration, and acculturation is intricate and multifaceted (20, 21). Migrants are exposed to new food environments, marketing strategies, and social norms surrounding eating, which can significantly influence their dietary choices; the degree of dietary change varies considerably among individuals and groups, depending on their level of integration into the host society, cultural preservation efforts, and access to resources that support healthy eating.

### Age and gender

Age and gender play significant roles in shaping dietary acculturation patterns among migrant populations, with younger migrants often exhibiting greater dietary shifts due to increased exposure to host-country food environments and social influences. Additionally, dietary acculturation can exhibit gender-specific patterns, with men and women potentially responding differently to migration-related factors. For example, among South Asian migrants in New Zealand, 63% of women reported a reduction in green leafy vegetable consumption post-migration, while 44% of men reported an increase in alcohol intake (22). In the United Kingdom, South Asian women’s fiber intake was reported to decrease by up to 40%, and alcohol intake among South Asian men increased from negligible levels to 15–25% after migration. Qualitative evidence from Syrian migrants in Germany supports these trends, with younger men adopting more Western food habits such as frequent fast food consumption, while women—especially those with children—are more likely to retain traditional cooking practices. Older migrants generally retain stronger ties to their traditional dietary practices, while younger individuals are more likely to adopt host-country dietary habits (13).

### Length of residence

Length of residence in the host country is a critical determinant of dietary acculturation, with longer durations typically associated with more pronounced dietary changes. Migrants who have resided in the host country for extended periods often exhibit greater assimilation into the prevailing food culture, leading to increased consumption of local foods and decreased reliance on traditional dietary staples. However, the relationship between length of residence and dietary habits is not always linear, as some studies have found that dietary changes may plateau or even reverse after a certain period. This could be due to a renewed emphasis on cultural identity and traditional practices, or to health concerns arising from prolonged exposure to less healthy host-country diets (18, 23–25).

### Socioeconomic status and education

Socioeconomic status (SES) and education are powerful determinants of dietary choices among migrant populations, influencing both access to healthy foods and the knowledge and resources necessary to make informed dietary decisions (2, 26). Migrant households with limited financial resources may face barriers to accessing fresh produce and nutritious foods, particularly in urban areas where healthy options may be more expensive and less readily available. Conversely, higher levels of education are generally associated with greater awareness of the health implications of dietary choices, as well as a greater capacity to navigate the complexities of the host-country food environment (27–29). Lower SES exacerbates unhealthy behaviors, especially when combined with loneliness and a lack of integration in the host country (25). Moreover, higher levels of education may empower migrants to critically evaluate dietary information and resist the influence of marketing tactics promoting unhealthy foods (2).

### Social environment and type of migration

The social environment and migration context exert a profound influence on dietary acculturation, shaping food choices and eating behaviors through social norms, cultural values, and access to social support networks (20, 21, 30). Migrants often encounter new social norms and expectations related to food, which can either promote or hinder the adoption of healthier dietary practices (2). For instance, migrants who settle in communities with strong ethnic enclaves may be more likely to maintain traditional dietary practices, while those who are more isolated may be more susceptible to the influences of the host-country food environment. Furthermore, the type of migration (e.g., economic migration, family reunification, refugee resettlement) can also impact dietary acculturation, as different migration contexts may be associated with varying levels of social support, economic opportunities, and exposure to cultural influences (23, 31). Migrants often grapple with adjusting to novel culinary landscapes and dietary norms in their adopted nations, which can lead to shifts in food preferences and consumption patterns (2).

### Food environment and access

The food environment plays a central role in shaping dietary acculturation, with the availability, affordability, and accessibility of various food options influencing dietary choices and eating behaviors among migrant populations. Migrants moving from low- and middle-income countries to high-income countries undergo a rapid shift from conventional eating habits to industrialized food options, causing negative health outcomes (3). Migrants who settle in areas with limited access to fresh produce and healthy food options may face significant barriers to maintaining a nutritious diet, while those who reside in food deserts may be more reliant on processed foods and fast food outlets. The food environment of the host country plays a significant role in modifying dietary patterns of immigrants (5). Supermarkets, farmers’ markets, and ethnic grocery stores can all influence dietary choices by providing access to a diverse range of food options, while food advertising and marketing can shape food preferences and consumption patterns (32). Food insecurity, busier lifestyles, and children’s preferences can also influence the dietary patterns of migrants (2). Changes in dietary behavior due to migration are likely to impact health (1, 2, 19, 20, 33). It is crucial to acknowledge that newcomers can acculturate into the host culture while retaining or not retaining their cultural roots (2). Food serves as a crucial strategy for integrating into a new environment, particularly for immigrants (34). The food environment and its intersection with cultural and socioeconomic variables are key determinants of dietary behavior among migrant populations.

### Impact on gut microbiota

Recent studies indicate that dietary acculturation leads to measurable changes in the gut microbiota of migrants (35). These changes may have significant implications for digestive health, immune function, and even mental well-being (4). The gut microbiota, a complex ecosystem of microorganisms residing in the digestive tract, plays a crucial role in human health, influencing everything from nutrient absorption and immune function to mental health and disease risk (36). Dietary shifts associated with migration, such as increased consumption of processed foods, saturated fats, and sugars, can disrupt the balance of the gut microbiota, leading to decreased diversity and an overgrowth of opportunistic pathogens (37). A diet high in fiber, fermented foods, and plant-based proteins promotes a diverse and resilient gut microbiota. As a defense mechanism, gut epithelial cells produce a mucosal barrier to segregate microbiota from host immune cells and reduce intestinal permeability. An impaired interaction between gut microbiota and the mucosal immune system can lead to an increased abundance of potentially pathogenic gram-negative bacteria and their associated metabolic changes, disrupting the epithelial barrier and increasing susceptibility to infections (37). The composition of the gut microbiota is intricately linked to dietary patterns, with specific dietary components promoting the growth of certain microbial species while suppressing others.

## Discussion

Our review of dietary transformations in migrant populations reveals several key findings. Across diverse groups, migration is frequently accompanied by a shift away from traditional, high-fiber dietary staples toward greater consumption of energy-dense, ultra processed foods (4). This transition is strongly influenced by acculturation level, socioeconomic status, and the surrounding food environment. The resulting dietary changes are consistently associated with higher prevalence of obesity, type 2 diabetes, cardiovascular diseases, and, in some groups, poorer mental health outcomes. These patterns and their associated health outcomes are summarized in Table 1, which provides an overview of the dietary changes, health risks, and modulating factors observed among the major migrant groups included in this review. Notably, the pace and extent of dietary acculturation—and the associated health risks—vary by age, gender, migration context, and the degree of social and cultural integration (20). These findings underscore the need for culturally tailored interventions that respect dietary preferences and traditions while addressing the broader determinants of health. This heterogeneity is detailed in Table 1, which illustrates how dietary changes and health outcomes differ according to population subgroup, geographic context, and influencing factors such as length of residence, socioeconomic status, and social support.

**Table 1:** Dietary acculturation in migrant populations: summary of key findings.

Population/group	Key dietary changes after migration	Health effects observed	Influencing factors/context	References
Immigrant Women	↑ Fat/sugar, ↓ fruits/vegetables, ↑ convenience foods, ↑ portions	↑ CVD, hypertension, diabetes (risk ↑ over time)	Busier lifestyle, stress, food insecurity, acculturation	(59, 60)
Hispanic Immigrants (US)	↑ Processed/Western diet with longer US stay	↑ BMI, mixed self-reported health	Acculturation, English at work, years in US	(61, 62)
General Immigrants	↑ Energy-dense foods, loss of traditional diet	Poorer mental health, ↑ chronic disease worry	SES, social exclusion, food insecurity	(63, 64)
Immigrants (US/Canada)	Diet quality declines over time, less healthy than at arrival	Declining health post-migration (“healthy immigrant effect” fades)	Access to traditional foods, family food choices	(53, 65)
African Immigrants (US)	Less acculturated: more traditional foods; more acculturated: ↑ fruits	Diabetes/hypertension risk varies by acculturation	Cultural preferences, social support, family influence	(66, 67)
Chinese Immigrants (US/Canada)	↑ Western food, inadequate fruit/veg/fiber intake	↑ Disease risk factors with acculturation	Demographics, family, community, cultural beliefs	(68, 69)
Immigrant Groups (US)	↑ “Festival foods” (high carb/fat/sugar)	↑ Cardiometabolic risk	Tradition, frequency of festival food	(70, 71)
South Asian Immigrants (Canada/UK)	↑ Adoption of Western dietary patterns, ↑ processed foods, ↓ traditional high-fiber staples	↑ Obesity and type 2 diabetes prevalence	Acculturation level, lack of culturally tailored assessment tools	(72, 73)
Ghanaian Immigrants (Europe)	Mixed maintenance of traditional staples with Western snacks and sweets	↑ Obesity and related cardiovascular risk	Food environment, affordability, dual food preferences	(74, 75)
Moroccan Immigrants (Netherlands)	Continued access to traditional foods, but increased convenience food use with time in host country	Mixed outcomes; some maintained diet diversity, others gained weight	Dynamic food environment, affordability, family influence	(1, 76, 77)
Sudanese Refugees (Australia)	↑ High-fat, processed food intake post-resettlement	↑ Obesity and type 2 diabetes risk	Food insecurity, Western food dominance in low-cost options	(78, 79)
Older Immigrants (e.g., Middle East→Europe/North America)	Retention of traditional food in home context, incorporation of host-country items in social/festival meals	≥ Mixed mental health outcomes, some positive identity reinforcement	Age at migration, cultural/family rituals, social integration	(12, 80, 81)

Addressing the complex and multifactorial nature of dietary transformation in migrant populations requires a collaborative, multisectoral approach (6). Policymakers, healthcare providers, researchers, and community organizations must work together to reduce barriers related to unemployment, low income, inadequate housing, and limited access to healthy foods (38). Effective interventions should be culturally sensitive, available in multiple languages, and designed with input from the communities they aim to serve. Community-based participatory research is particularly valuable for engaging migrants in the development, implementation, and evaluation of nutrition interventions (39). Nutrition education programs should focus on building practical skills and knowledge, and can be delivered through community centers, schools, workplaces, and healthcare settings. Public health initiatives must address both individual behavior and social context, recognizing the powerful influence of family, social networks, and community norms on food choices (40).

Technology-enabled approaches, such as mobile apps, telehealth, and SMS-based interventions, offer new opportunities to deliver accessible, personalized nutrition support (41). Community-based programs—including cooking classes, gardening, and food assessments—can foster social connection and empower migrants to make healthier choices (42). Policy-level actions, such as subsidies for healthy foods, taxes on unhealthy products, regulations on food marketing, and improved food labeling, are critical to create supportive food environments and address systemic barriers (43). It is also essential to enhance clinician knowledge about culturally relevant nutrition and the importance of food in disease prevention. Ongoing monitoring and evaluation, supported by robust research and adequate funding, will ensure that interventions remain effective and responsive to evolving needs (44).

Improving dietary habits and health outcomes for migrants also means understanding and addressing the broader psychosocial challenges many face, including acculturative stress, discrimination, and social isolation (20). Mental health support, culturally sensitive counseling, and peer-led programs can complement nutrition interventions and address interconnected health needs. Interventions targeting migrant mothers or other family influencers may be particularly effective in shaping household dietary practices. Structural measures such as food labeling, restricting marketing of unhealthy foods to children, and ensuring access to healthy retailers are necessary complements to individual-focused strategies. Initiatives like community gardens and farmers’ markets can increase access to fresh produce, foster engagement, and build resilience at the local level (45–47).

Healthcare access and patient-provider communication are additional priorities. Migrants often have limited health literacy, face language barriers, or lack familiarity with host-country healthcare systems. Training clinicians in cultural competence and employing interpreters or bilingual staff can help bridge these gaps, as can providing health information in multiple languages and tailored to cultural context. Policy frameworks should ensure that healthcare for migrants is affordable, equitable, and responsive to diverse needs. Active involvement of community health workers and family members further supports effective care and outreach (48).

Despite these advances, many migrant health policies still overlook lifestyle-related risk factors and preventive care. A comprehensive approach, integrating culturally appropriate health promotion, routine migration history assessment, and awareness of cultural health practices, is needed to address inequities in access and outcomes. Effective interventions should be linguistically and culturally appropriate, address administrative and legal barriers, and ensure ongoing coordination and funding. Given the continued rise in global migration, addressing the health needs of migrants is an urgent public health priority. Policies should recognize the diversity of migrant experiences, ethical imperatives for equity, and the benefits of investing in the health and well-being of all communities (38, 49, 50).

## Limitations and future directions

Research on dietary transformations and health implications in migrant populations faces several limitations that need to be addressed in future studies. One major challenge is the heterogeneity of migrant populations, as migrants come from diverse cultural, socioeconomic, and geographical backgrounds. This heterogeneity makes it difficult to generalize findings from one migrant group to another. Studies often lack longitudinal data, making it challenging to track dietary changes and health outcomes over time and to establish causal relationships. Future research should focus on using longitudinal designs to understand the long-term effects of dietary transformations on the health of migrant populations. Many studies rely on self-reported dietary data, which are subject to recall bias and social desirability bias, thus, future studies should incorporate more objective measures of dietary intake, such as biomarkers and dietary records, to improve the accuracy of data collection. There is a need for more culturally sensitive research methods that account for the unique experiences and perspectives of migrant populations (11).

Community-based participatory research approaches, which involve collaboration with community members in all stages of the research process, can be particularly valuable for ensuring that research is culturally relevant and responsive to community needs (39). Qualitative research methods, such as focus groups and in-depth interviews, can provide valuable insights into the lived experiences of migrant populations and the factors that influence their dietary choices. Future research should explore the complex interplay of factors that contribute to dietary transformations in migrant populations, including socioeconomic status, cultural norms, access to healthcare, and the food environment (20). Additionally, there is a need for more intervention studies that evaluate the effectiveness of different strategies for promoting healthy eating among migrant populations.

Furthermore, future research should address the gaps in our understanding of the dietary transformations and health implications in specific migrant groups, such as refugees and undocumented immigrants. Given the increasing number of refugees worldwide, there is a pressing need for research that examines the unique nutritional challenges faced by this population (51). The intersection of these factors can create unique challenges for refugees in accessing healthy foods and maintaining traditional dietary practices. Acculturation processes can lead to the abandonment of traditional diets and the adoption of less healthy eating patterns. In addition, future research should explore the role of the food environment in shaping dietary choices among migrant populations.

More research is needed to understand how these factors influence dietary choices and health outcomes in migrant populations. Understanding how migration and acculturation affect dietary habits, especially among Latino immigrants, is crucial (21). Studies on Mexican immigrant women suggest that dietary acculturation is complex, highlighting the importance of considering cultural aspects in designing strategies to improve their diet and health (52). Longitudinal studies are needed to monitor dietary habits over time and understand how they change in response to acculturation and other factors (53). There is a need to develop and validate culturally appropriate tools for assessing dietary intake and acculturation in migrant populations (6). Given that dietary acculturation is specific to the culture and country of origin, it is important to define the population group when examining measures of dietary acculturation (6).

Sub-Saharan African immigrants in Western countries have not been studied extensively regarding changes in their eating habits. It is important to conduct further research on migration trends, dietary habits, and dietary acculturation of Sub-Saharan African immigrants living in the United States, the United Kingdom, and France (54). More studies are needed to understand the factors that promote healthy dietary choices and prevent chronic diseases in migrant populations (6, 13, 54, 55). Comprehensive dietary and health screenings, culturally appropriate nutrition education resources, and interventions are needed to improve refugees’ dietary intake and nutritional status (51). Dietary habits are critical for preventing and treating chronic diseases, but the role of diet in immigrant transitions is not well understood (19). Migrants often experience significant changes in their dietary habits as they adapt to new environments (56). The availability of familiar foods, affordability, and cultural influences all play a role in shaping their food choices. More research is needed to understand how these factors interact to affect the health of migrant populations. Effective strategies are needed to promote healthy eating and prevent chronic diseases in migrant populations.

## Conclusion

Migration is a multifaceted phenomenon that profoundly influences dietary habits and health outcomes, and dietary acculturation, the process by which migrants adopt the dietary practices of their host country, plays a pivotal role in these transformations (26). Migrants often experience a shift in their dietary patterns as they navigate new food environments, socioeconomic conditions, and cultural norms (1). These dietary changes can have both positive and negative consequences for their health, increasing the risk of chronic diseases such as obesity, type 2 diabetes, and cardiovascular disease. The effect of demographic and socio-cultural variables on changes in food habits after migration should also be considered (57). For instance, obesity levels were observed to be elevated among African migrants residing in England compared to those in other European countries (58). The extent of dietary change varies depending on individual circumstances and the degree of integration into the host society.

Addressing the nutritional challenges faced by migrant populations requires a multi-pronged approach that considers the complex interplay of factors that influence their dietary choices. Promoting culturally sensitive nutrition education programs, improving access to healthy and affordable foods, and creating supportive social environments are essential steps in fostering healthy eating habits among migrants. Future research should focus on developing and evaluating interventions that are tailored to the specific needs and cultural backgrounds of different migrant groups. By understanding the dietary transformations and health implications in migrant populations, we can develop effective strategies to promote their health and well-being, ensuring that they have the opportunity to thrive in their new environments.
